# Amphiphilic Protein Surfactants Reduce the Interfacial Tension of Biomolecular Condensates

**DOI:** 10.1021/acs.langmuir.5c03118

**Published:** 2025-08-27

**Authors:** Bruna Favetta, Huan Wang, Zheng Shi, Benjamin S. Schuster

**Affiliations:** † Department of Biomedical Engineering, 242612 Rutgers, The State University of New Jersey, Piscataway, New Jersey 08854, United States; ‡ Department of Chemistry and Chemical Biology, Rutgers, The State University of New Jersey, Piscataway, New Jersey 08854, United States; § Department of Chemical and Biochemical Engineering, Rutgers, The State University of New Jersey, Piscataway, New Jersey 08854, United States

## Abstract

Biomolecular condensates are protein-dense regions in cells that often arise from liquid–liquid phase separation. Interfacial tension is a key determinant of biomolecular condensate behavior, influencing condensate size and interactions with intracellular structures. Certain proteins and RNAs are known to selectively localize to the interface of condensates, where they can regulate condensate function in cells. Previously, we designed amphiphilic proteins that preferentially adsorb to the surface of condensates. These proteins contain one condensate-philic domain (RGG) and one condensate-phobic domain (MBP or GST). Here, we demonstrate through direct quantification that these amphiphilic proteins act as surfactants, reducing the interfacial tension of RGG–RGG condensates from ∼260 to ∼100 μN/m in a concentration-dependent manner. Notably, the GST-based surfactant protein exhibits a 10-fold greater efficacy in lowering interfacial tension compared with the MBP-based surfactant. We show that this increased efficacy is due to its higher surface density, driven by GST’s ability to oligomerize. We also show that these surfactant proteins slow droplet fusion and reduce the average droplet size, as would be expected of a typical surfactant. Our findings quantitatively show how surfactant proteins can play a critical role in regulating the behavior of biomolecular condensates by modulating their interfacial tension.

## Graphical abstract

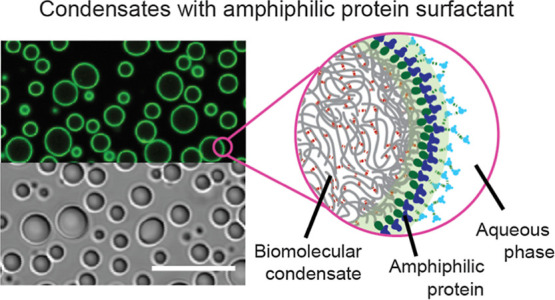

## Introduction

Biomolecular condensates are membraneless organelles that often arise from liquid–liquid phase separation, organizing key cellular processes such as transcription, signal transduction, and stress response.^1,2^ The physical properties of these condensates, including their interfacial tension, play a critical role in determining their size, miscibility, dynamics, and interactions with other cellular structures.^3,4^ For example, the different interfacial tensions of nucleolar condensates determine the multiphase architecture of the nucleolus.^5^ The interfacial tension of DNA–protein condensates can affect the rate of gene expression.^6^ Also, the degree of condensate wetting, partly determined by the interfacial tension of the condensate, regulates the process of autophagosome formation.^7^

The interfacial tension of biomolecular condensates arises from the balance of intermolecular interactions between proteins and their surrounding environment.^3^ Inside the condensate, attractive interactions, such as hydrogen bonding, hydrophobic interactions, and electrostatic forces between amino acids, promote cohesion among proteins.^8^ At the interface with the surrounding medium, these interactions are reduced, leading to an energetic penalty for exposing certain residues or domains to the surrounding environment. This energetic penalty creates a tension at the interface, acting to minimize the interfacial area of condensates.^9^ Because proteins are macromolecules^10^ and because protein–protein interactions in condensates are often polar and thus similar in nature to those in the aqueous dilute phase,^8^ the interfacial tension of condensates is low compared to that of more commonly studied emulsions, such as oil–water.^11^ Still, due to this interfacial tension, condensates coarsen over time, either through Ostwald ripening or droplet coalescence.^12,13^ The distribution of condensate size is thought to be regulated in cells^14–16^ and has been shown to be important for condensate function.^17,18^

One mechanism that has been proposed to be used by cells to control condensate size is surfactant-like proteins.^19^ Surfactants are molecules that lower interfacial tension and kinetically stabilize emulsions.^20^ Several proteins that interact with condensates in cells have been suggested to have surfactant-like features. During mitosis, the proliferation marker protein Ki-67 prevents chromosomes from merging into a single chromatin mass.^21^ However, whether Ki-67 affects the interfacial tension has not been measured. In a second example, the protein NO145 preferentially partitions to the interface of *Xenopus laevis* nucleoli and may prevent fusion of nucleoli.^14^ In a third example, MLX, a transcription regulator, preferentially locates to the surface of condensates composed of the transcription factor TFEB in vitro and lowers the interfacial tension of TFEB condensates by around 2.5 times, thus decreasing their affinity for a specific DNA motif.^22^ However, the mechanism of regulation of the interfacial tension was not explored. In general, factors that determine a condensate’s interfacial tension are poorly understood. Consequently, developing model surfactant proteins and examining how they influence condensates can provide valuable insights into the underlying mechanisms of condensate interfacial tension regulation in cells.

In previous work, we designed amphiphilic proteins containing an intrinsically disordered region (IDR) fused to folded domains.^19^ We utilized the soluble folded domain glutathione *S*-transferase (GST) or maltose binding protein (MBP), which we fused to the intrinsically disordered RGG domain of the P granule protein LAF-1. This RGG domain has been previously shown to be necessary and sufficient for LAF-1 phase separation.^23^ RGG is a 168-residue domain enriched in Arg, Gly, Tyr, and uncharged polar residues.^24^ Two amphiphilic protein constructs we designed previously and further characterized here are MBP–GFP–RGG and GST–GFP–RGG.^19^ At low concentrations, these amphiphilic proteins form a film that coats the surface of a condensate composed of the RGG–RGG protein (a tandem repeat of the RGG domain). This can be understood as follows: in the amphiphilic fusion proteins, the RGG domain is “condensate-philic”, thus preferring to interact with the RGG–RGG condensate. The folded MBP or GST domains are “condensate-phobic”, thus preferring to interact with the dilute phase. We included GFP to visualize the localization of the amphiphilic proteins.

Here, we directly measure the effect of these amphiphilic proteins on the interfacial tension of RGG–RGG condensates. Using micropipette aspiration, we find that both amphiphilic proteins reduce condensate interfacial tension from approximately 260 to approximately 100 μN/m in a concentration-dependent manner. However, the GST-based surfactant is ∼10 times more efficient compared with the MBP-based surfactant. Furthermore, we show that the efficiency of interfacial tension reduction depends on the ability of amphiphilic proteins to form oligomers. The effect of amphiphilic proteins on condensate interfacial tension can be rationalized by a model in which increased amphiphilic protein density at the condensate surface directly reduces the interfacial tension. Consistent with their ability to reduce condensate interfacial tension, we observe that amphiphilic proteins slow fusion and reduce the size of condensates. Collectively, our results establish a quantitative relationship between the presence of surfactant proteins and condensate interfacial tension, shedding light on how cells regulate several aspects of condensate functions.

## Experimental Section

### Cloning

All genes of interest were cloned into pET vectors in frame with C-terminal 6×-His tags. RGG–RGG was cloned as previously described.^25^ Amphiphilic proteins were cloned as described previously.^19^ The RGG domain used here is the N-terminal IDR (residues 1–168) of *Caenorhabditis elegans* P granule protein LAF-1.^23^ Gene sequences were verified by Sanger sequencing (Azenta). Protein sequences are available in Supporting Information Note 1.

### Protein Expression and Purification

For bacterial expression, plasmids were transformed into BL21­(DE3) competent*Escherichia coli* (New England BioLabs). Colonies picked from fresh plates were grown for 8 h at 37 °C in 1 mL of LB while shaking at 250 rpm. This starter culture was then used to inoculate 0.5 L cultures. For GST–MBP–RGG and MBP–GFP–RGG, cultures were grown in 2 L baffled flasks in Terrific Broth medium (Fisher Scientific) supplemented with 4 g/L glycerol while being shaken at 250 rpm. The flasks were shaken at 37 °C until the OD_600_ reached approximately 1, at which time expression was induced with 500 μM isopropyl β-d-1-thiogalactopyranoside (IPTG), and the temperature was reduced to 18 °C for overnight expression. For RGG–RGG, cultures were grown in 2 L baffled flasks in autoinduction medium (Formedium) supplemented with 4 g/L glycerol at 37 °C overnight while being shaken at 250 rpm. The pET vectors used contained a kanamycin resistance gene; kanamycin was used at concentrations of 50 μg/mL in cultures. After overnight expression at 18 or 37 °C, bacterial cells were pelleted by centrifugation at 4100*g* at 4 °C. Pellets were resuspended in lysis buffer (1 M NaCl, 20 mM Tris, 20 mM imidazole, Roche EDTA-free protease inhibitor, pH 7.5) and lysed by sonication. Lysate was clarified by centrifugation at 25,000*g* for 30 min at 25 °C. The clarified lysate was then filtered with a 0.22 μm filter. Lysis was conducted on ice, but other steps were conducted at room temperature to prevent phase separation.

Proteins were purified using an AKTA Pure FPLC with 1 mL of nickel-charged HisTrap columns (Cytiva) for immobilized metal affinity chromatography of the His-tagged proteins. After injecting proteins onto the column, the column was washed with 500 mM NaCl, 20 mM Tris, and 20 mM imidazole, pH 7.5. Proteins were eluted with a linear gradient of imidazole up to 500 mM NaCl, 20 mM Tris, and 500 mM imidazole, pH 7.5. Proteins were dialyzed overnight using 7 kDa MWCO membranes (Slide-A-Lyzer G2, Thermo Fisher) into physiological buffer (150 mM NaCl and 20 mM Tris, pH 7.5), with the exception that GST–GFP–RGG was dialyzed into high salt buffer (500 mM NaCl and 20 mM Tris, pH 7.5). Proteins were dialyzed at room temperature (20 °C) except for RGG–RGG, which was dialyzed at 42 °C to inhibit phase separation because the phase-separated protein bound irreversibly to the dialysis membrane. Proteins were snap frozen in liquid nitrogen in single-use aliquots and stored at −80 °C.

### Confocal Microscopy

Protein samples were prepared as follows: RGG–RGG protein aliquots were thawed at 42 °C, above its phase transition temperature. MBP–GFP–RGG and GST–GFP–RGG aliquots were thawed at room temperature. Protein concentrations were measured based on their absorbance at 280 nm using a NanoDrop spectrophotometer (ThermoFisher); RGG–RGG was mixed in a 1:1 volumetric ratio with 8 M urea to prevent phase separation during concentration measurements.

First, RGG–RGG and the buffer were mixed at room temperature. Then, amphiphilic proteins MBP–GFP–RGG and GST–GFP–RGG were added at the desired protein concentrations. The final buffer conditions were 150 mM NaCl and 20 mM Tris, pH 7.5. Protein samples were plated on 16-well glass-bottom dishes (1.5 glass thickness; Grace Bio-Labs) that were coated with 5% Pluronic F-127 (Sigma-Aldrich) for a minimum of 10 min to create a hydrophilic surface and prevent condensates from wetting the glass. The chambers were washed with water prior to plating the protein samples.

Confocal imaging was performed on a Zeiss Axio Observer 7 inverted microscope equipped with an LSM900 laser scanning confocal module and employing a 63×/1.4 NA plan-apochromatic, oil-immersion objective. GFP was excited to fluoresce with a 488 nm laser. Confocal fluorescence images were captured by using GaAsP detectors. Transmitted light images were collected with either the ESID module or an Axiocam 702 sCMOS camera (Zeiss), in both cases using a 0.55 NA condenser.

### Droplet Image Analysis

Image analysis and data processing were performed in MATLAB version R2024b. The fluorescence intensity profile of the condensates with GFP-tagged amphiphilic proteins was measured by using the Circular Hough Transform (imfindcircles function) to identify droplet locations and by drawing a line that spanned the droplet diameter plus 25% of the radius length in each direction across the droplets. Condensate size was also calculated using the Circular Hough Transform (imfindcircles function).

### Micropipette Aspiration (MPA)

The micropipette aspiration (MPA) experiments were carried out on a Ti2-A inverted fluorescence microscope (Nikon, Japan), with a 60× water objective (NA 1.2), a Hamamatsu camera (ORCA-Fusion, Flash4.0 V3, Hamamatsu), equipped with a motorized stage and two motorized 4-axes micromanipulators (PatchPro-5000, Scientifica) and a multitrap optical tweezers (Tweez305, Aresis, Slovenia) according to the protocol we reported previously^11,26^ with minor modifications.

Micropipettes were made by pulling glass capillaries using a pipette puller (PUL-1000, World Precision Instruments). The pipette tip was then cut to achieve an opening diameter ranging from 2 to 5 μm. Subsequently, the pipette was bent to an angle of approximately 40° using a microforge (DMF1000, World Precision Instruments) so that the tip of the micropipette would be parallel to the imaging plane. The micropipette was filled with the same buffer as used in other microscopy experiments (150 mM NaCl, 20 mM Tris, pH 7.5) using a MICROFIL needle (World Precision Instruments). The filled micropipette was then mounted onto a micromanipulator. The rear end of the pipette was connected to an automatic pressure controller (Flow-EZ, Fluigent; pressure resolution 1 Pa). The MPA experiments were conducted in glass-bottom dishes (D35-20-1.5-N, Cellvis), under bright-field illumination to minimize potential artifacts associated with fluorescence excitation. Fluorescence images were taken after MPA experiments to confirm the presence of the core–shell structure. Typically, optical tweezers-assisted condensate fusion was first carried out to achieve a large (diameter >5 μm) condensate for accurate MPA measurements. To minimize sample evaporation, 1.5 mL of Milli-Q water was added to the edge of the dish (separate from the sample), and the dishes were covered with a thin plastic wrap with a ∼2 mm hole for pipette insertion. Zero pressure of the aspiration pipette was calibrated before each set of experiments by determining the pressure at which small condensates underwent Brownian motion inside the micropipette.

Measurement of condensate viscosity was carried out as described in Wang et al.^11^ and analyzed according to the protocol described in Roggeveen et al.^26^ Briefly, normalized aspiration length (aspiration length, *L*_p_, over the pipette radius, *R*_p_) was measured over time for each pressure step using ImageJ v1.54. For each step, the slope of a linear fitting of (*L*_p_/*R*_p_)^2^ vs time is equal to the effective shear rate. Then, the slope of the aspiration pressure vs shear rate graph gives 4η, where η is viscosity. Under all conditions where condensate viscosity values were measured, the condensate always wet the inner wall of the micropipette. In principle, the intercept is the interfacial tension of the condensate. In our case, measurements would need to be taken infinitely slowly to account for equilibration of the surfactant, so the intercept was not used to measure the interfacial tension.

Instead, to more accurately quantify the interfacial tension of condensates, a static tension measurement protocol was used.^27,28^ A large condensate was drawn into the micropipette by a small suction pressure (typically ∼5 Pa), and then, the pressure was increased stepwise until the deformation of the condensate interface was equal to the inner radius of the micropipette (Supporting Information Appendix, Figure S1). During this process, images were collected at each pressure step. The radii of the condensate protrusion and external condensate droplet were measured for each pressure step using ImageJ (version 2.1). For each step, the interfacial tension, γ, was calculated using the equation^27^

1 $$\gamma =\frac{P_{asp}}{2\left[\left(\frac{1}{R_{i}}\right)-\left(\frac{1}{R_{0}}\right)\right]}$$

where *R*_i_ is the radius of curvature of the condensate interface inside the pipette, *R*_0_ is the radius of the condensate outside the pipette, and *P*_asp_ is the aspiration pressure used. Interfacial tensions measured from each step were averaged such that all pressure steps received the same weight to obtain the interfacial tension of the condensate.

### Optical Tweezers-Assisted Droplet Fusion

An optical tweezers-assisted fusion assay was carried out and analyzed following the protocol previously described.^11^ Two condensates were independently trapped with minimal laser power. The fusion events were captured at 20 Hz. MATLAB R2024b was used to fit the contour of the fusing condensates to an ellipse. The fusion time (τ) was found by fitting the relaxation in aspect ratio (AR; defined as the ratio between the long and short axis of the ellipse) over time (*t*) to a stretched exponential decay,^29^ according to the following empirical equation:

2 $$AR=1+\left({AR}_{0}-1\right)\;e^{-{\left(t/\tau \right)}^{1.5}}$$

where AR_0_ is the aspect ratio at time 0, defined as the start of the droplet fusion event.

## Results and Discussion

### Amphiphilic Proteins Reduce the Interfacial Tension of RGG–RGG Condensates

In agreement with our previous results, both amphiphilic proteins tested, MBP–GFP–RGG and GST–GFP–RGG, form a film surrounding an RGG–RGG condensate core when 10 μM RGG–RGG is mixed with 1 μM amphiphilic protein (Figure 1A–C).

**Figure 1:**
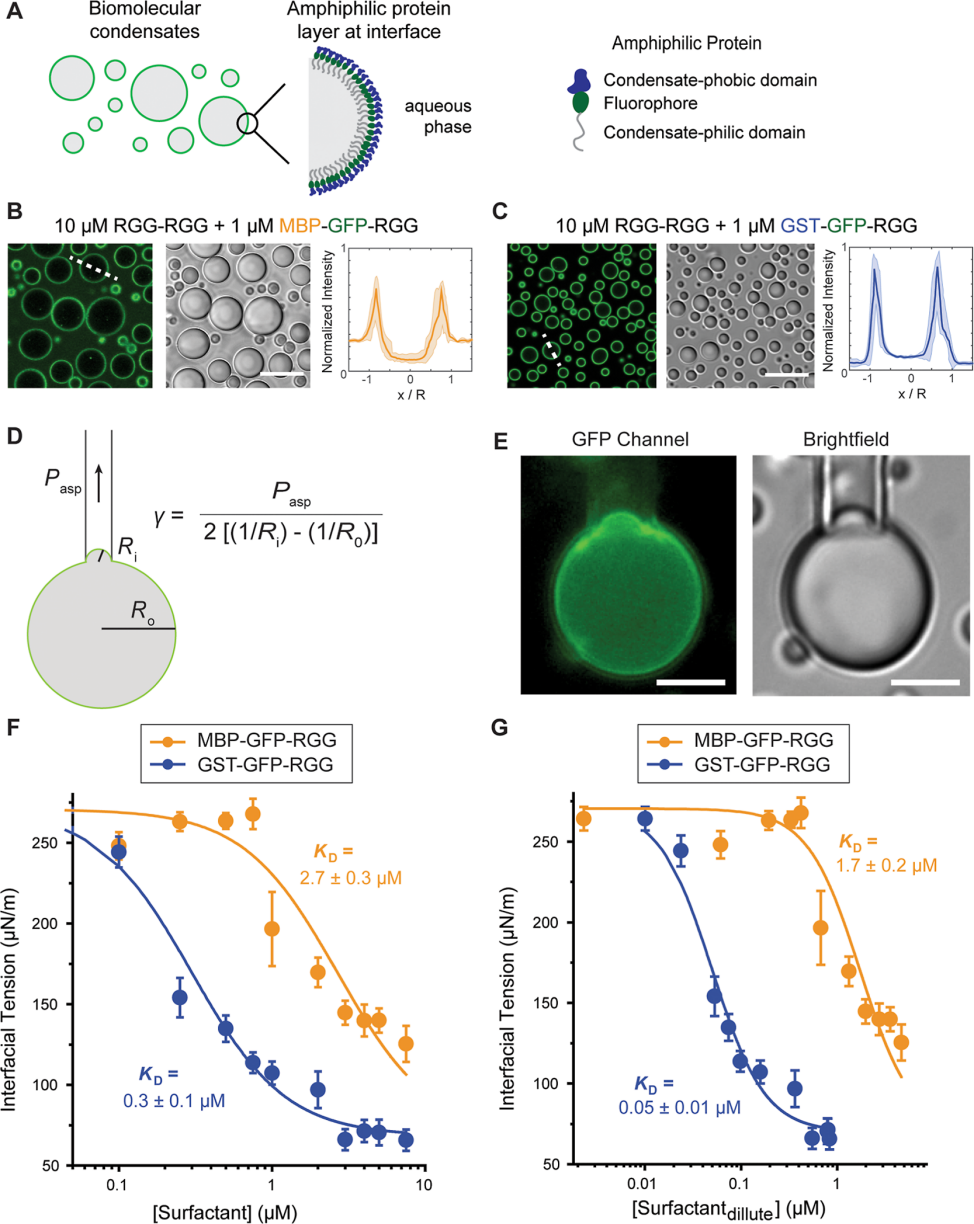
Amphiphilic proteins lower condensate interfacial tension. (A) Schematic model of the engineered amphiphilic proteins localized on the surface of an RGG–RGG condensate. (B,C) Transmitted light and confocal fluorescence imaging of core–shell condensates formed by mixing 10 μM RGG–RGG and 1 μM MBP–GFP–RGG or GST–GFP–RGG. Fluorescence intensities were quantified as line profiles across individual condensates, normalized by condensate size. Intensities were also divided by 65,535, the dynamic range of the images, such that intensities scale from 0 to 1. *N* > 50 condensates, from images taken from three independent samples. The shaded area represents 1 standard deviation. (Scale bars, 10 μm.) (D) Schematic representation of the experimental setup for measuring interfacial tension using micropipette aspiration and the corresponding equation. (E) Widefield imaging of condensate aspiration experiment, showing that partially aspirated condensate retains amphiphilic protein layer at interface (scale bars, 5 μm). (F) Interfacial tension of RGG–RGG condensates measured with increasing concentrations of either amphiphilic protein, MBP–GFP–RGG or GST–GFP–RGG. (G) Interfacial tension of RGG–RGG condensates measured against the equilibrium dilute phase concentration of either amphiphilic protein. The lines in F and G represent fitting curves using the Hill equation (eq 3; *R* ^2^ = 0.98 and 0.94 for MBP–GFP–RGG and GST–GFP–RGG, respectively, in F; *R* ^2^ = 0.98 and 0.94 for MBP–GFP–RGG and GST–GFP–RGG, respectively, in G). Error bars indicate ±1 standard error of the mean.

To measure the interfacial tension of condensates with amphiphilic proteins, we use Micropipette Aspiration (MPA), as described in Figure 1D,E and the Experimental Section Micropipette Aspiration (MPA). We found that both amphiphilic proteins behave as surfactants and reduce the interfacial tension of RGG–RGG condensates in a surfactant-concentration-dependent manner (Figure 1F) while minimally impacting the viscosity of the condensates (Supporting Information Appendix, Figure S2). For MBP–GFP–RGG, at concentrations up to 0.75 μM, we observe a plateau in the interfacial tension measurements at ∼260 μN/m. As the concentration of MBP–GFP–RGG continues to increase, the interfacial tension of condensates significantly decreases and reaches ∼125 μN/m at 7.5 μM amphiphilic protein. For GST–GFP–RGG, a significant decrease in interfacial tension is observed with much smaller concentrations of the amphiphilic protein, as low as 0.25 μM. With increasing GST–GFP–RGG concentrations, we observe a reduction in interfacial tension until reaching a plateau at ∼65 μN/m.

We next sought to quantitatively define the interfacial effects of each surfactant protein. Given the sigmoidal shape of the interfacial tension (γ) vs surfactant concentration (*C*) curves, the traditional Szyszkowski–Langmuir equation cannot fully capture the trend of the data (Supporting Information Appendix, Figure S3). We therefore fit our results to the Hill equation,^30^ assuming that the density of surfactants adsorbed to the interface of condensates linearly reduces the interfacial tension (Supporting Information Appendix Note 2)­

3 $$\gamma =\gamma_{0}-\frac{\gamma_{0}-\gamma_{\infty }}{1+{\left(\frac{K_{D}}{C}\right)}^{p}}$$

Here, *C* is the surfactant concentration, γ_0_ and γ_∞_ are the initial and final interfacial tension, and *K*_D_ and *p* are the dissociation constant and cooperativity coefficient of the interfacial adsorption of the surfactants, respectively. To minimize fitting uncertainty, we carried out a global fit to both the MBP–GFP–RGG and GST–GFP–RGG data with γ_0_, γ_∞_, and *p* as shared parameters and only *K*_D_ as a free fitting parameter. We find that for MBP–GFP–RGG, *K*_D_ = 2.7 ± 0.3 μM, whereas for GST–GFP–RGG, *K*_D_ = 0.3 ± 0.1 μM. Therefore, GST–GFP–RGG is 9 times more efficient than MBP–GFP–RGG in reducing the interfacial tension of RGG–RGG condensates (Figure 1F and Supporting Information Appendix, Figure S4). We also found *p* = 1.6 ± 0.3, suggesting a cooperative binding mechanism for surfactant proteins, perhaps because binding of surfactant molecules induces ordering of RGG–RGG molecules at the interface, which promotes the adsorption of additional surfactant molecules.^31^

Interestingly, our results indicate a correlation between the brightness of the fluorescence at the condensate interface and the efficiency of the amphiphilic protein in reducing interfacial tension. As noted above, we can observe the degree of protein adsorption to the condensate surface by confocal fluorescence imaging. We measured the degree of partitioning of MBP–GFP–RGG and GST–GFP–RGG in the dilute phase, interface, and condensed phase (Figure 1B,C). Fluorescence intensities were quantified as line profiles across individual condensates normalized by condensate size. Intensities were also divided by 65,535, the dynamic range of the 16-bit images, such that intensities scale from 0 to 1. At 1 μM amphiphilic protein, the average partitioning for MBP–GFP–RGG is 0.26 ± 0.02 : 0.70 ± 0.09 : 0.11 ± 0.03 in the dilute phase, interface, and condensed phase, respectively. For GST–GFP–RGG, we found an average partitioning of 0.04 ± 0.01 : 0.89 ± 0.02 : 0.14 ± 0.03, respectively. Therefore, the GST-based amphiphilic protein prefers the interface more than the MBP-based amphiphilic protein. The increased partitioning to the condensate surface by the GST-based amphiphilic protein likely contributes to its greater strength as a surfactant, an observation that we further explore in Figure 2.

**Figure 2:**
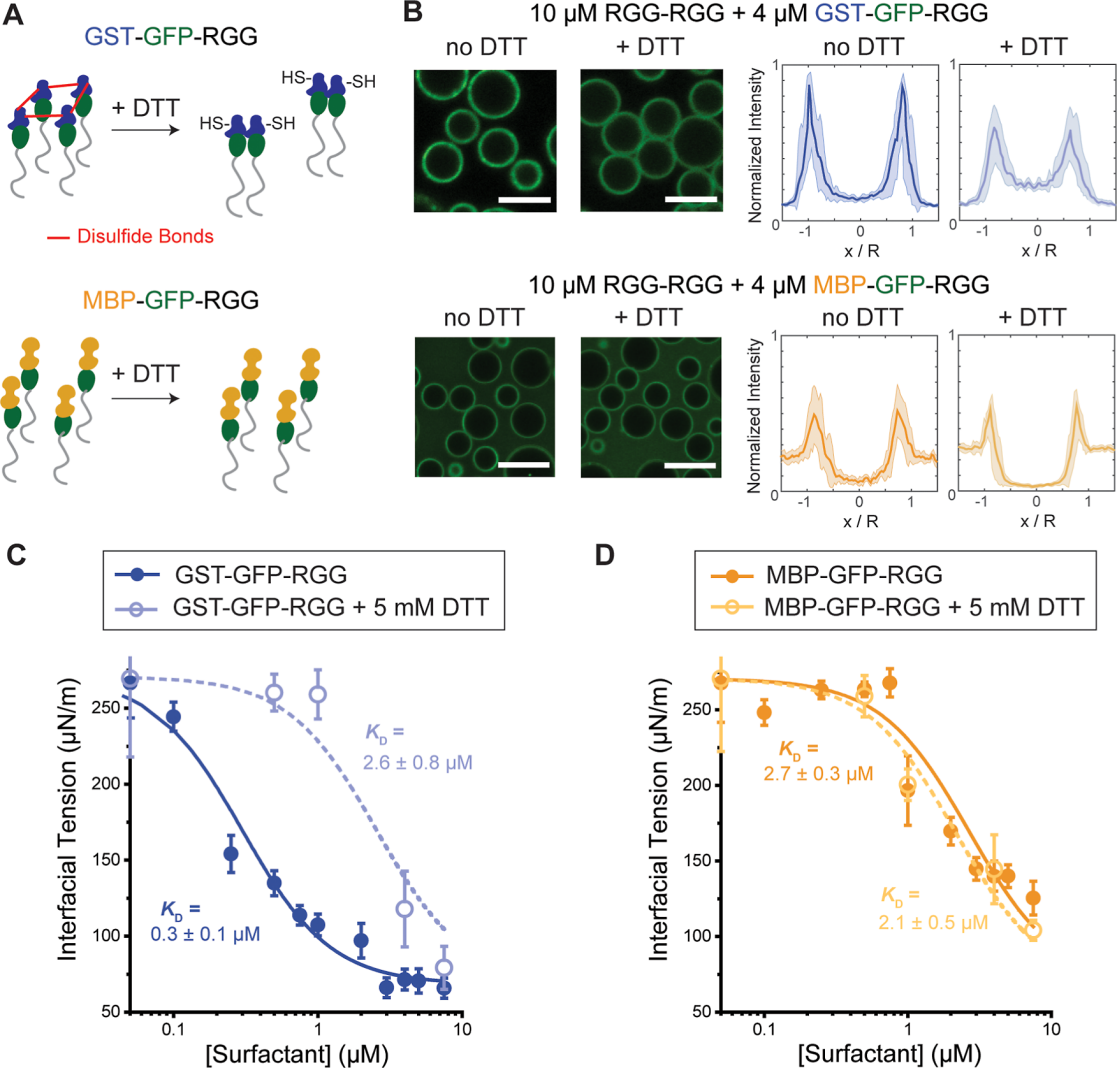
Modulation of surfactant interfacial behavior using reducing agent DTT. (A) Schematic depicting that DTT inhibits disulfide bond formation between cysteines in the GST domains, while not affecting MBP. (B) Confocal fluorescence imaging of the condensates shows adding 5 mM DTT lowered the partitioning of GST–GFP–RGG to the interface, with no effect on MBP–GFP–RGG (scale bars, 5 μm). Fluorescence intensities were quantified as line profiles across individual condensates, normalized by condensate size. Intensities were also divided by 65,535, the dynamic range of the images, such that intensities scale from 0 to 1. *N* > 20 condensates, from images taken from two independent samples. The shaded area represents 1 standard deviation. The effect of GST–GFP–RGG (C) and MBP–GFP–RGG (D) on condensate interfacial tension with and without the addition of DTT. The lines represent fitted curves to the Hill equation (*R* ^2^ = 0.94 and 0.98 for the dashed curve and solid curve, respectively, in C; *R* ^2^ = 0.94 and 0.96 for the dashed curve and solid curve, respectively, in D). Error bars indicate 1 standard error of the mean.

The partitioning analysis above also indicates that there is a difference between the equilibrium dilute phase concentrations of samples with GST–GFP–RGG and MBP–GFP–RGG. This suggests that there may be a difference between the equilibrium dilute phase concentrations and the total concentration initially added of the amphiphilic proteins. To account for this effect, we generated a standard curve relating GFP fluorescence intensity to the molar concentration of each amphiphilic protein, using the same buffer solutions as in our experiments (Supporting Information Appendix, Figure S5). We then used the standard curve along with our confocal images of condensates to determine the equilibrium concentration of amphiphilic proteins in the dilute phase for each concentration tested, allowing us to replot the interfacial tensions as a function of equilibrium surfactant concentrations in the dilute phase (Figure 1G). Using this approach, the difference in *K*_D_ between MBP–GFP–RGG and GST–GFP–RGG increases from 9 times to 34 times, further supporting our finding that the GST–GFP–RGG amphiphile is more efficient at reducing interfacial tension.

### Oligomerization Increases Surfactant Strength

Next, we investigated the cause of the difference in the surfactant strength between the two amphiphilic proteins. Unlike MBP, which is monomeric and lacks cysteines, GST is dimeric and can form oligomers due to its four cysteine residues (per monomer), three of which are highly solvent exposed and can easily form disulfide bonds.^32^ These disulfide bonds can be reduced by dithiothreitol (DTT) (Figure 2A). Therefore, we tested the effect of DTT on the partitioning of surfactant proteins and their ability to decrease the interfacial tension. After adding 5 mM DTT, the partitioning of GST–GFP–RGG to the interface of RGG–RGG condensates was reduced, compared to no observable change for the partitioning of MBP–GFP–RGG (Figure 2B). For example, in samples with 4 μM GST–GFP–RGG, the partitioning of amphiphilic protein shifted from 0.09 ± 0.02 : 0.90 ± 0.08 : 0.16 ± 0.04 in the dilute phase, interface, and condensed phase, respectively, without DTT, to 0.10 ± 0.03 : 0.58 ± 0.08 : 0.24 ± 0.06 with DTT, which represents a 35% reduction in partitioning to the interface.

To assess whether DTT affects the amphiphilic proteins’ ability to decrease condensate interfacial tension, we repeated our MPA measurements with the addition of 5 mM DTT (Figure 2C,D). Again, we fit our data to the Hill equation (eq 3) and carried out a global fit to both the MBP–GFP–RGG and the GST–GFP–RGG data with γ_0_, γ_∞_, and *p* as shared parameters and only *K*_D_ as a free fitting parameter. We found that DTT has a negligible effect on the interfacial tension of RGG–RGG condensates with the MBP–GFP–RGG surfactant (*K*_D_ = 2.7 ± 0.3 μM without DTT and 2.1 ± 0.5 μM with DTT). However, DTT strongly impairs the surfactant activity of GST–GFP–RGG, with *K*_D_ increasing upon adding DTT from 0.3 ± 0.1 μM to 2.6 ± 0.8 μM, which is comparable to that of MBP–GFP–RGG. Thus, our findings suggest that the ability of GST to oligomerize and form disulfide bonds drives its higher partitioning to the condensate surface and increases its strength as a surfactant. In prior work, we showed that the strength of interaction between “condensate-phobic” domains partially determines the degree of partitioning of an amphiphilic protein to the surface of a condensate.^19^ Here, we found that the greater partitioning of GST-based amphiphiles to the surface of condensates also correlates with a greater reduction in interfacial tension. Our findings also suggest that modulation of the redox environment is one mechanism that can be used to regulate the strength of a protein surfactant, which we speculate may be relevant in cells.

### Amphiphilic Proteins Slow Droplet Fusion and Reduce Condensate Size

Droplet coalescence is driven by interfacial tension. We therefore asked whether surfactants increase condensate fusion time. Here, we utilized an optical tweezers-assisted droplet fusion assay to quantify the fusion time and the inverse capillary velocity of condensates at various concentrations of amphiphilic proteins. As shown in Figure 3A and quantified in Figure 3B, we observed that the addition of either amphiphilic protein increased condensate fusion time. We fit the merging condensates to a stretched exponential equation to quantify the change in aspect ratio over time.^29^ Two ∼4 μm radius RGG–RGG condensates fused within 0.4 s, whereas condensates of similar sizes in samples with 5 μM MBP–GFP–RGG fused within 0.8 s, and those with 5 μM GST–GFP–RGG fused in 2 s.

**Figure 3:**
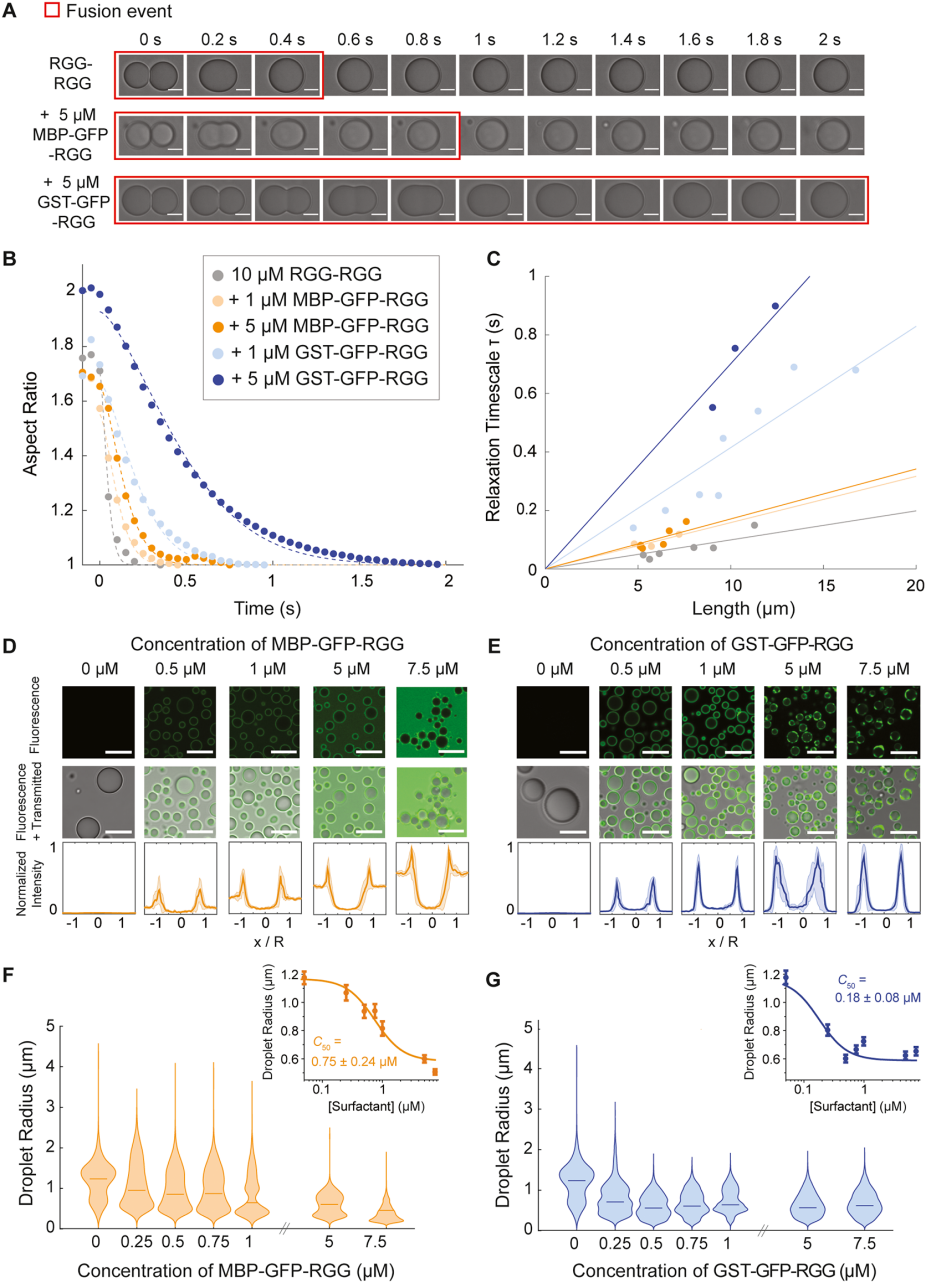
Amphiphilic proteins slow droplet fusion and also reduce the average size of condensates. (A) Time-lapse brightfield images of optical tweezers-assisted fusion of condensates, with and without the amphiphilic proteins. Red boxes represent the fusion events, where the aspect ratio changes from ∼2 to 1. Scale bars, 5 μm. (B) Relaxation of condensate aspect ratios over time with and without the addition of amphiphilic protein, comparing similarly sized (∼4 μm radii) condensates. Increasing the concentration of amphiphilic proteins slows the relaxation of the aspect ratio. *R* ^2^ is 0.99 for 10 μM RGG–RGG, 0.99 for +1 μM MBP–GFP–RGG, 0.99 for +5 μM MBP–GFP–RGG, 0.99 for +1 μM GST–GFP–RGG, and 0.99 for +5 μM GST–GFP–RGG. (C) Relaxation time scale of droplet fusion, plotted against droplet length (the geometric mean of condensate diameters before fusion). The lines are linear fits for each condition. *R* ^2^ is 0.72 for 10 μM RGG–RGG, 0.69 for +1 μM MBP–GFP–RGG, 0.64 for +5 μM MBP–GFP–RGG, 0.82 for +1 μM GST–GFP–RGG, and 0.86 for +5 μM GST–GFP–RGG. (D,E) Transmitted light and confocal fluorescence imaging of the condensates formed by mixing RGG–RGG (10 μM) with different concentrations of MBP–GFP–RGG (D) and GST–GFP–RGG (E). Droplet radii become smaller as amphiphilic protein concentration increases. Fluorescence intensities were quantified as line profiles across individual condensates, normalized by condensate size. Intensities were also divided by 65,535, the dynamic range of the images, such that intensities scale from 0 to 1. *N* > 50 condensates, from images taken from three independent samples. The shaded area represents 1 standard deviation. Scale bars, 5 μm. (F,G) Violin plots depicting shift in size of condensates with increasing concentrations of MBP–GFP–RGG (F) and GST–GFP–RGG (G). Bars indicate the median droplet size in each condition. Inset, the relationship between average condensate sizes and the concentration of either amphiphilic protein, MBP–GFP–RGG or GST–GFP–RGG, fit to a sigmoidal equation (eq 5). *C*_50_, the concentration required to reduce average condensate radius by half, is noted (*R* ^2^ is 0.93 for F, 0.81 for G). Error bars indicate 1 standard error of the mean.

Next, we extracted relaxation time scales and plotted those against condensate radius for multiple fusion events (Figure 3B,C) to obtain the inverse capillary velocity, η/γ

4 $$\frac{\eta }{\gamma }\approx \frac{\tau }{l}$$

where η is the viscosity, γ is the interfacial tension, τ is the relaxation time scale, and l is the condensate length determined as the geometric mean of the condensate diameters prior to fusion.^11,29^ Our results indicate that increasing concentrations of amphiphilic protein increase the slope and therefore the inverse capillary velocity of samples. For RGG–RGG, we find an inverse capillary velocity of 0.010 s/μm, compared to 0.016 and 0.017 s/μm for samples with 1 and 5 μM MBP–GFP–RGG, versus 0.041 and 0.070 s/μm for samples with 1 and 5 μM GST–GFP–RGG. We can also calculate inverse capillary velocity from our micropipette aspiration data, from which we determined viscosity (Supporting Information Appendix, Figure S2) and interfacial tension (Figure 1). From our micropipette aspiration data, we obtain inverse capillary velocities of 0.014 s/μm for RGG–RGG, 0.025 for samples with 1 μM MBP–GFP–RGG and 0.047 for samples with 1 μM GST–GFP–RGG. Comparing the results, we find agreement between the inverse capillary velocities calculated from the fusion data compared to that from the micropipette aspiration data (Supporting Information Appendix, Table S1). Therefore, using the concept of inverse capillary velocity, we can connect the increase in droplet fusion time to the reduction in interfacial tension in samples with amphiphilic proteins, noting that the surfactant proteins did not change condensate viscosity.

Interestingly, we also noted cases with >5 μM amphiphilic protein concentration where we could not induce droplet fusion with our optical tweezers (Supporting Information Appendix, Movie S1). This result suggests that the amphiphilic protein film at the surface of condensates acts as a barrier that significantly hinders condensate fusion beyond the expected effect of lowered interfacial tension. Therefore, we next tested whether the amphiphilic proteins affect the condensate size distribution. For this, we measured droplet sizes in samples with a wide range of amphiphilic protein concentrations. As expected, both amphiphilic proteins preferentially locate to the surface of condensates composed of RGG–RGG, within the range of concentrations tested (Figure 3D,E). With increasing concentrations of MBP–GFP–RGG, we observe an increase in fluorescence at both the condensate interface and the aqueous phase with little change in fluorescence inside condensates. For GST–GFP–RGG, an increase in protein concentration caused an increase in fluorescence signal mainly at the condensate interface, including phase separation of GST–GFP–RGG itself at the interface at the concentrations ≥5 μM, in agreement with our earlier study.^19^ No inhomogeneities in GST–GFP–RGG fluorescence can be visually observed at the condensate surface with lower surfactant protein concentrations (Supporting Information Appendix, Figure S6); under conditions in which GST–GFP–RGG phase separates (concentration ≥5 μM), bright fluorescent puncta can be observed at the interface along with a continuous layer of surfactant protein that remains surrounding the entire condensate interface (Supporting Information Appendix, Figure S7).

We studied droplet sizes at the 1 h time point (Figure 3F,G). We observed a shift to smaller droplet sizes with increasing concentration for both amphiphilic proteins. Without the addition of amphiphilic proteins, we observed a median droplet radius of 1.2 μm. Addition of high concentrations of either amphiphilic protein results in samples with condensates of a median radius of 0.5–0.6 μm. We fit our data to a sigmoidal equation, which was selected empirically, as it captures the shape of the data and allows for comparison with eq 3

5 $$r=r_{0}-\frac{r_{0}-r_{\infty }}{1+{\left(\frac{C_{50}}{C}\right)}^{n}}$$

Here, *C* is the surfactant concentration, *r*_0_ and *r*_∞_ are the initial and final average droplet radii, respectively, *C*_50_ is the concentration required to reduce the average condensate radius by half, and *n* is the Hill coefficient. We find that for MBP–GFP–RGG, *C*_50_ = 0.75 ± 0.24 μM, and for GST–GFP–RGG, *C*_50_ = 0.18 ± 0.08 μM. The difference in magnitude between these two fitting parameters is smaller than those measured for the condensate interfacial tension. This is likely due to condensate size being regulated by additional factors beyond interfacial tension; for instance, surface charge may contribute to kinetic stability of condensates and other emulsions.^33^ Notably, at high surfactant concentrations (>5 μM), we observe droplets that remain in contact for long times without fusing, as mentioned previously (Supporting Information Appendix, Movie S1). This is possibly because an adsorbed surfactant protein monolayer can create strong electrostatic and steric repulsion that inhibits droplet coalescence, much like classical chemical surfactants.^34^ This surfactant protein layer may also be viscoelastic and mechanically impede fusion. In summary, our results suggest that surfactant proteins reduce the droplet size.

## Conclusions

In this study, we quantify how engineered amphiphilic proteins modulate biomolecular condensate interfacial tension. Our results directly demonstrate that the amphiphilic proteins act as surfactants and reduce the interfacial tension of condensates. We show that the degree of surfactant protein partitioning to the condensate surface determines its strength in lowering the interfacial tension. By modulating the redox environment and thus preventing disulfide cross-linking between GST–GFP–RGG proteins, we can significantly reduce both the surfactant’s adsorption to the condensate interface and its capacity to lower interfacial tension. Furthermore, we also found that the reduction in interfacial tension results in slower fusion kinetics and contributes to reduced condensate sizes.

Our results reveal emerging design principles for protein-based surfactants that localize to condensate interfaces. Although our insights were developed using RGG-based proteins, we believe these principles are broadly applicable to other systems composed of biomolecular condensates and amphiphilic proteins. In previous work, we used coarse-grained molecular dynamics simulations to study the behavior of amphiphilic proteins composed of two domains: X (condensate-phobic) and A (condensate-philic).^19^ The behavior of this amphiphilic X–A protein was simulated in the presence of a condensate composed of A–A protein. The simulations showed that successful interfacial localization of X–A requires a balance of interactions: domain X must not interact too strongly with A, or else the amphiphilic protein partitions into the condensate interior instead of accumulating at the interface; increasing the strength of X self-interaction favors X–A partitioning to the interface. Building on this, in the current work, we now compare the surfactant activity of two amphiphilic proteins, with different X domains but the same A domain, that both localize to the A–A condensate interface. We find that when X has a stronger tendency to self-interact, the amphiphilic protein accumulates more densely at the interface, acting as a more effective surfactant for reducing the interfacial tension. In our experiments, oligomerization of the X domain enhanced surface localization and led to a greater reduction in the condensate interfacial tension. Based on this, we speculate that other strategies to promote surfactant surface density may similarly enhance surfactant activity.

This work, together with other recent research,^17,19^ suggests that protein-based surfactants may be one of several methods used to regulate the interfacial tension of condensates in cells. Given the importance of interfacial tension in determining the behavior of condensates,^3^ these surfactant proteins may have extensive impacts on the function of condensates. For example, they may affect condensate multiphasic architecture, interaction with other intracellular structures, or size. Furthermore, we speculate that cells may control the strength of surfactants, such as by modulating the redox environment with the presence of reducing agents or using other mechanisms such as post-translational modification of proteins.

Looking ahead, in bioengineering applications, amphiphilic proteins such as those described here can be used to improve engineered condensate systems. For example, engineered condensates have been explored as the basis for in vitro biocatalysis systems.^35,36^ The ability to optimize biocatalysis in condensates by controlling the condensate size, condensate surface area to volume ratio, and thus mass transfer rates should be explored in the future.

## Supplementary Material





## Data Availability

Requests for further information and resources should be directed to and will be fulfilled by Benjamin S. Schuster (benjamin.schuster@rutgers.edu) or Zheng Shi (zheng.shi@rutgers.edu).

## Notes

Protein sequences and equation derivations; additional results supporting the inverse capillary velocity data; and images of the experimental setup and additional results related to the viscosity of condensates, alternative fitting models and partial fits, and the homogeneity and continuity of the surfactant layer (PDF)
Additional fusion experiment results (AVI)
