# Supplementary material for: Amphiphilic Protein Surfactants Reduce the Interfacial Tension of Biomolecular Condensates
Source: Langmuir. 2025 Aug 27;41(35):23827–36. doi: 10.1021/acs.langmuir.5c03118 (PMC12424167; doi:10.1021/acs.langmuir.5c03118)
Supplement: Supplementary file 1 [file la5c03118_si_001.pdf]

Supporting Information for

Amphiphilic protein surfactants reduce the interfacial tension of biomolecular condensates

Bruna Favetta[1,‡], Huan Wang[2,‡], Zheng Shi[2,*], Benjamin S. Schuster[3,*]

1 Department of Biomedical Engineering, Rutgers, The State University of New Jersey, Piscataway, NJ 08854, USA

2 Department of Chemistry and Chemical Biology, Rutgers, The State University of New Jersey, Piscataway, NJ 08854, USA

3 Department of Chemical and Biochemical Engineering, Rutgers, The State University of New Jersey, Piscataway, NJ 08854, USA

‡ These authors contributed equally.

* Corresponding authors: zheng.shi@rutgers.edu and benjamin.schuster@rutgers.edu

This PDF file includes:

Supplementary Notes 1 – 2
Supplementary Table
Figures S1 – S7
Caption SI Movie 1

Supplementary Notes

1. Protein sequences used in this work

Throughout this paper, RGG denotes the RGG domain from LAF-1 (residues 1-168 of LAF-1). All proteins included a hexahistidine tag at the C-terminus for immobilized metal affinity chromatography. Below, RGG, GST, MBP, and GFP domains are color-coded; tags, linkers, and cut sites are not colored.

RGG-RGG

MESNQSNNGGSGNAALNRGGRYVPPHLRGGDGGAAAAASAGGDDRRGGAGGGGYRRGGGN

SGGGGGGGYDRGYNDNRDDRDNRGGSGGYGRDRNYEDRGYNGGGGGGGNRGYNNNRGG

GGGGYNRQDRGDGGSSNFSRGGYNNRDEGSDNRGSGRSYNNDRRDNGGDGEFGKLMESNQ

SNNGGSGNAALNRGGRYVPPHLRGGDGGAAAAASAGGDDRRGGAGGGGYRRGGGNSGGGG

GGGYDRGYNDNRDDRDNRGGSGGYGRDRNYEDRGYNGGGGGGGNRGYNNNRGGGGGGY

NRQDRGDGGSSNFSRGGYNNRDEGSDNRGSGRSYNNDRRDNGGDGLEHHHHHH

MBP-GFP-RGG

MKIEEGKLVIWINGDKGYNGLAEVGKKFEKDTGIKVTVEHPDKLEEKFPQVAATGDGPDIIFWAHDR

FGGYAQSGLLAEITPDKAFQDKLYPFTWDAVRYNGKLIAYPIAVEALSLIYNKDLLPNPPKTWEEIPAL

DKELKAKGKSALMFNLQEPYFTWPLIAADGGYAFKYENGKYDIKDVGVDNAGAKAGLTFLVDLIKNK

HMNADTDYSIAEAAFNKGETAMTINGPWAWSNIDTSKVNYGVTVLPTFKGQPSKPFVGVLSAGINA

ASPNKELAKEFLENYLLTDEGLEAVNKDKPLGAVALKSYEEELVKDPRIAATMENAQKGEIMPNIPQ

MSAFWYAVRTAVINAASGRQTVDEALKDAQTNSSSNNNNNNNNNNLGETVRFQSMVSKGEELFT

GVVPILVELDGDVNGHKFSVSGEGEGDATYGKLTLKFICTTGKLPVPWPTLVTTLTYGVQCFSRYPD

HMKQHDFFKSAMPEGYVQERTIFFKDDGNYKTRAEVKFEGDTLVNRIELKGIDFKEDGNILGHKLEY

NYNSHNVYIMADKQKNGIKVNFKIRHNIEDGSVQLADHYQQNTPIGDGPVLLPDNHYLSTQSKLS
KDPNEKRDHMVLLEFVTAAGITLGMDELYKGGGSENLYFQGEFGKLMESNQSNNGGSGNAALNR
GGRYVPPHLRGGDGGAAAAASAGGDDRRGGAGGGGYRRGGGNSGGGGGGGYDRGYNDNRD
DRDNRGGSGGYGRDRNYEDRGYNGGGGGGGNRGYNNNRGGGGGGYNRQDRGDGGSSNFS
RGGYNNRDEGSDNRGSGRSYNNDRRDNGGDGLEHHHHHH

GST-GFP-RGG

MSPILGYWKIKGLVQPTRLLLEYLEEKYEEHLYERDEGDKWRNKKFELGLEFPNLPYYIDGDVKLTQS
MAIIRYIADKHNMLGGCPKERAEISMLEGAVLDIRYGVSRIAYSKDFETLKVDFLSKLPEMLKMFEDR
LCHKTYLNGDHVTHPDFMLYDALDVVLYMDPMCLDAFPKLVCFKKRIEAIPQIDKYLKSSKYIAWPL
QGWQATFGGGDHPPGSETVRFQSMVSKGEELFTGVVPILVELDGDVNGHKFSVSGEGEGDATYG
KLTLKFICTTGKLPVPWPTLVTTLTYGVQCFSRYPDHMKQHDFFKSAMPEGYVQERTIFFKDDGNYK
TRAEVKFEGDTLVNRIELKGIDFKEDGNILGHKLEYNYNSHNVYIMADKQKNGIKVNFKIRHNIEDG
SVQLADHYQQNTPIGDGPVLLPDNHYLSTQSKLSKDPNEKRDHMVLLEFVTAAGITLGMDELYKG
GGSENLYFQGEFGKLMESNQSNNGGSGNAALNRGGRYVPPHLRGGDGGAAAAASAGGDDRR
GGAGGGGYRRGGGNSGGGGGGGYDRGYNDNRDDRDNRGGSGGYGRDRNYEDRGYNGGGG
GGGNRGYNNNRGGGGGGYNRQDRGDGGSSNFSRGGYNNRDEGSDNRGSGRSYNNDRRDN
GGDGLEHHHHHH

2. Hill equation derivation

Assuming that interfacial tension decreases linearly with the density of surfactant protein adsorbed to the interface of condensates, then:

$$\gamma = \gamma_0 - \alpha \cdot \rho_{\mathrm{s}} \tag{S1}$$

where γ is interfacial tension, γ_0 is the interfacial tension without surfactant protein, α is a constant, and ρ_{s} is the density of surfactant protein adsorbed to the interface of condensates.

We propose that surfactant binding to the condensate interface can be modeled using a Hill equation:

$$\rho_{\mathrm{s}} = \frac{\rho_{\max}}{1+\left(\frac{K_{\mathrm{D}}}{c}\right)^{p}} \tag{S2}$$

where $\rho_{\max}$ is the maximum density of surfactant protein at the condensate interface, c is the surfactant concentration, and K_{D} and p are the dissociation constant and cooperativity coefficient of the interfacial adsorption of the surfactants, respectively.

If we define $\gamma_0 - \gamma_\infty = \alpha\rho_{\max}$, then:

$$\gamma = \gamma_0 - \frac{\gamma_0 - \gamma_\infty}{1+\left(\frac{K_{\mathrm{D}}}{c}\right)^{p}} \tag{S3}$$

Supplementary Table

Table 1: Inverse capillary velocities calculated from fusion data and micropipette aspiration data

Protein Sample	Inverse Capillary Velocity from Fusion Experiments (s/μm) ± SEM	Inverse Capillary Velocity from Micropipette Aspiration Experiments (s/μm) ± SEM	Percent Difference (%)
10 μM RGG-RGG	0.010 ± 0.001	0.014 ± 0.001	33
10 μM RGG-RGG + 1 μM MBP-GFP-RGG	0.016 ± 0.002	0.025 ± 0.003	44
10 μM RGG-RGG + 5 μM MBP-GFP-RGG	0.017 ± 0.004	N/A	N/A
10 μM RGG-RGG + 1 μM GST-GFP-RGG	0.041 ± 0.003	0.047 ± 0.004	14
10 μM RGG-RGG + 5 μM GST-GFP-RGG	0.070 ± 0.007	N/A	N/A

Supplementary Figures

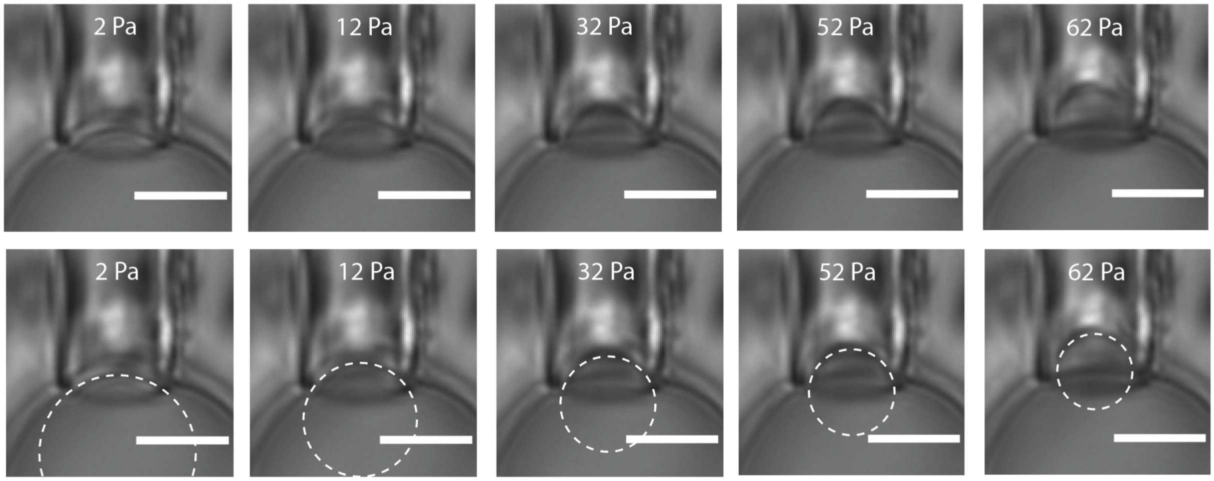

Figure S1: Stepwise aspiration of an RGG-RGG condensate with 4 µM GST-GFP-RGG. (Top) In 10 Pa increments, the condensate is gradually aspirated into the pipette until the deformation of the condensate interface is approximately equal to the inner radius of the micropipette. (Bottom) Dashed line indicates the fit to the radius of curvature of the deformed portion of the condensate. Scale bars, 5 µm.

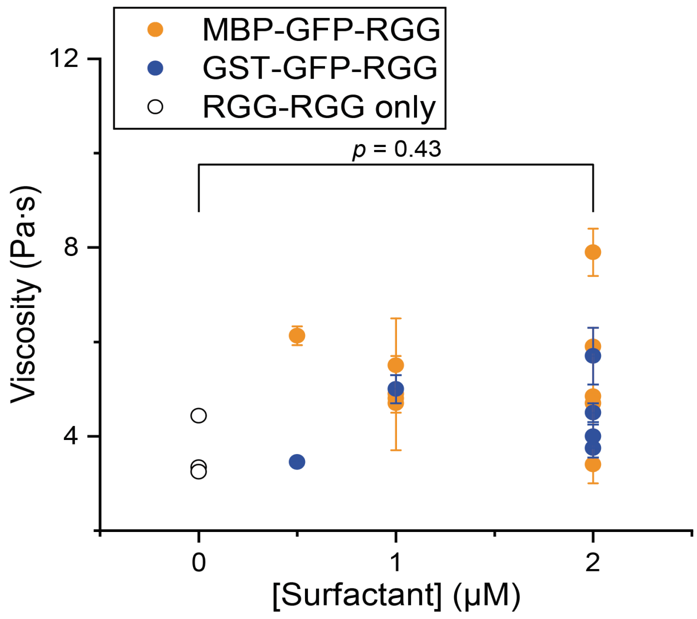

Figure S2: Dependence of condensate viscosity on the concentration of surfactant protein added. Each point shows the viscosity measured by micropipette aspiration for a single condensate, with error bars indicating the standard error for that measurement. Addition of surfactant does not significantly alter condensate viscosity; *p* values were determined using one-way ANOVA followed by post hoc Tukey's test.

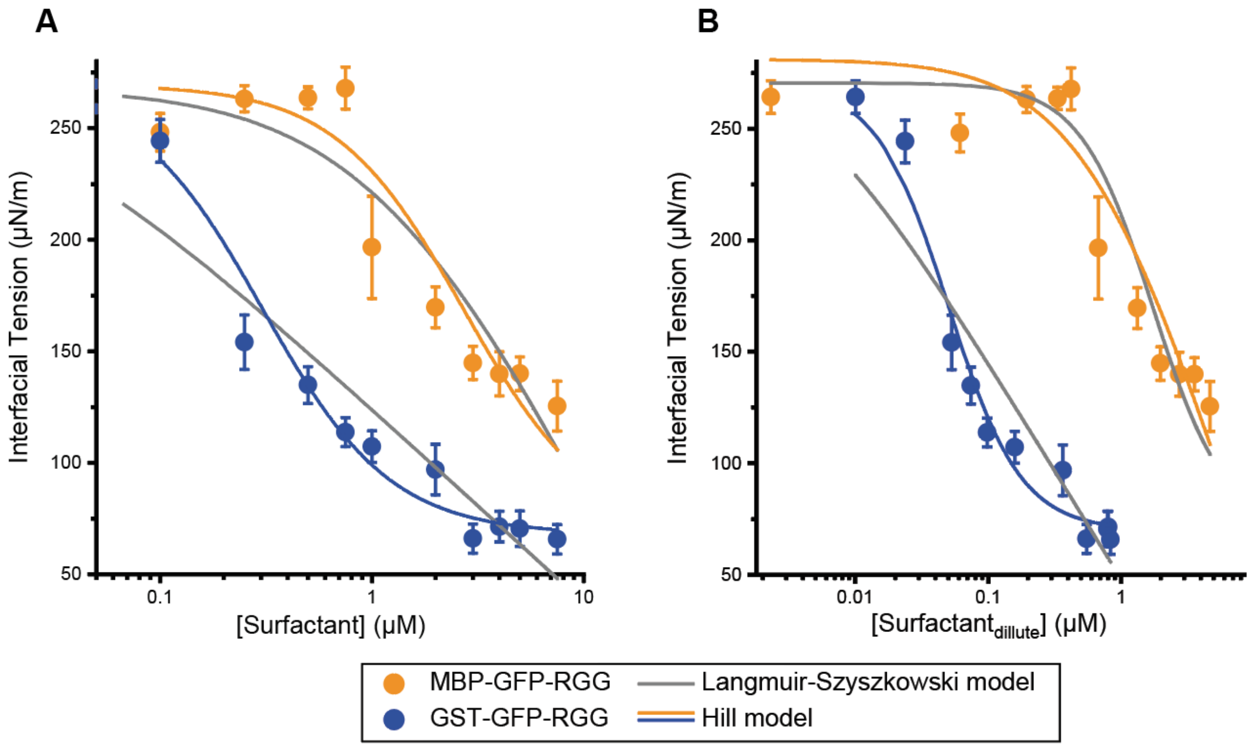

Figure S3: Fitting of interfacial tension data with two adsorption models. A) Interfacial tension of RGG-RGG condensates with increasing total concentrations of either amphiphilic protein. B) Interfacial tension of RGG-RGG condensates plotted against the equilibrium concentration of either amphiphilic protein in the dilute phase. The colored lines in panels A and B represent fitting curves using the Hill model (Eq. S3). Three fitting parameters (γ_0, γ_∞ and *p*) are shared for the two amphiphilic proteins, while K_D is determined per protein (resulting in two parameters), meaning there are a total of 5 fitting parameters. The gray lines represent fitting curves from the Langmuir-Szyszkowski model: $\gamma = \gamma_0 - B * \ln(1 + KC)$, $B = RT\Gamma_\infty$ where γ_0 is the initial interfacial tension, *R* is the gas constant, *T* the absolute temperature, Γ_∞ is the maximum possible adsorption at saturation, *C* is the surfactant concentration at equilibrium and K is the adsorption equilibrium constant. The fitting parameter γ_0 is shared for the two amphiphilic proteins, while *B* and *K* are determined per protein (resulting in four parameters), meaning that the

fit also uses a total of 5 fitting parameters. Although both models have 5 fitting parameters, the R^2 for the Hill model is 0.99 and 0.97 for panels A and B, respectively, while the R^2 for the Langmuir-Szyszkowski model is 0.94 and 0.92 for panels A and B, respectively.

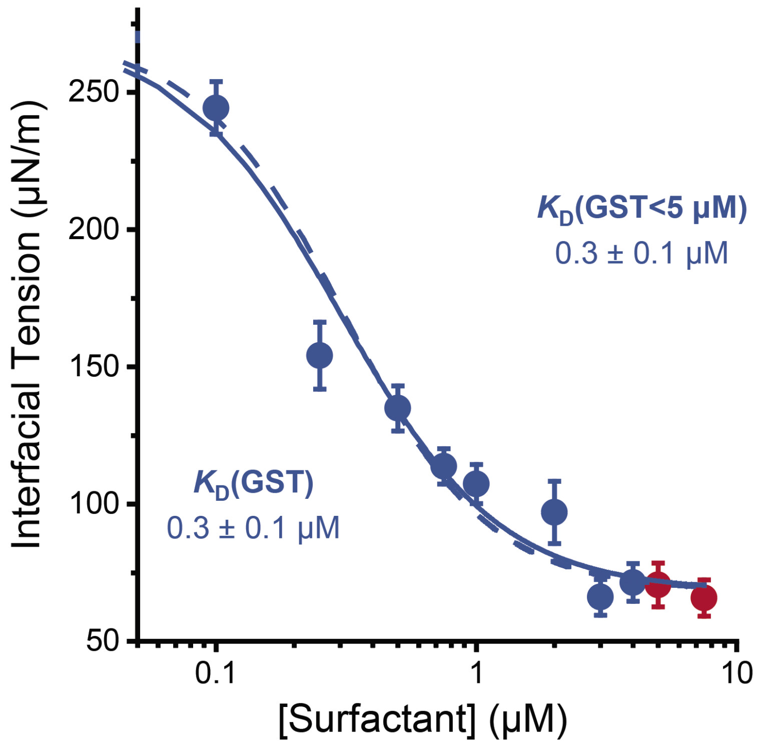

Figure S4: **Interfacial tension of RGG-RGG condensates measured with increasing concentrations of GST-GFP-RGG surfactant.** Fits using the Hill equation were conducted using the full data set compared to a truncated data set without [Surfactant] ≥ 5 μM (red data points, corresponding to conditions at which GST-GFP-RGG phase separates), showing that the K_D does not change. R^2=0.94 for both curve fits.

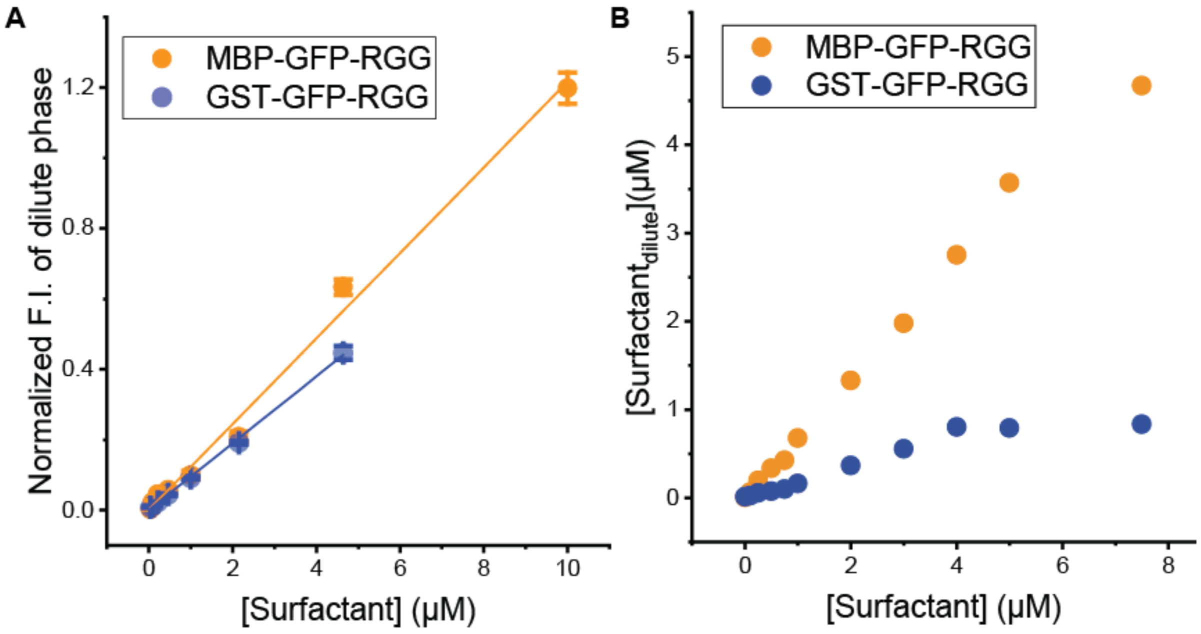

Figure S5: Quantification of the amount of amphiphilic protein that remains in the dilute phase. A) The calibration curves for MBP-GFP-RGG and GST-GFP-RGG. The relation between the normalized fluorescence intensity and the bulk surfactant concentration added to the sample (without RGG-RGG). Each point is the mean ± s.d. of five independent images; solid lines are linear fits used as calibration curves for converting fluorescence to absolute concentration (MBP-GFP-RGG: slope $= 0.121 \pm 0.004\ \mu M^{-1}$, R^2=0.98; GST-GFP-RGG: slope $= 0.095 \pm 0.001\ \mu M^{-1}$, R^2=0.99). B) Surfactant concentration in the dilute phase in samples with RGG-RGG, calculated using the equation from the calibration in A. MBP-based surfactant (orange) accumulates in the dilute phase more readily than the GST-based surfactant (blue) over the entire concentration range.

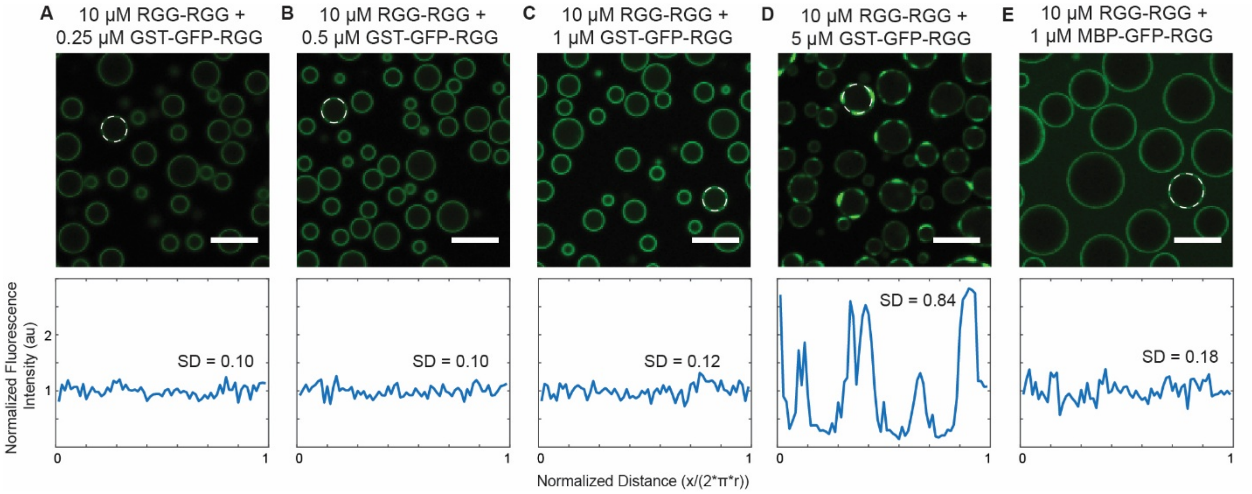

Figure S6: Surfactant protein forms a homogenous layer at low surfactant protein concentrations. (Top) Representative fluorescence images of samples with 10 µM RGG-RGG and a range of concentrations of GST-GFP-RGG (A, 0.25; B, 0.5; C, 1; and D, 5 µM), compared to a sample with 10 µM RGG-RGG and 1 µM MBP-GFP-RGG (E). Images with low concentrations of GST-GFP-RGG show no visually discernable inhomogeneities at the condensate surface, while phase separation is observed at 5 µM. Scale bars, 5 µm. (Bottom) Representative fluorescence intensity profiles around condensate circumference, from n = 1 condensate indicated by the white dashed line. Fluorescence values were normalized by the average fluorescence intensity of each condensate circumference. The standard deviation (SD) is noted for each data set; SD is low with GST-GFP-RGG concentration between 0.25 and 1 µM and increases more than five-fold when GST phase separates at the interface at 5 µM.

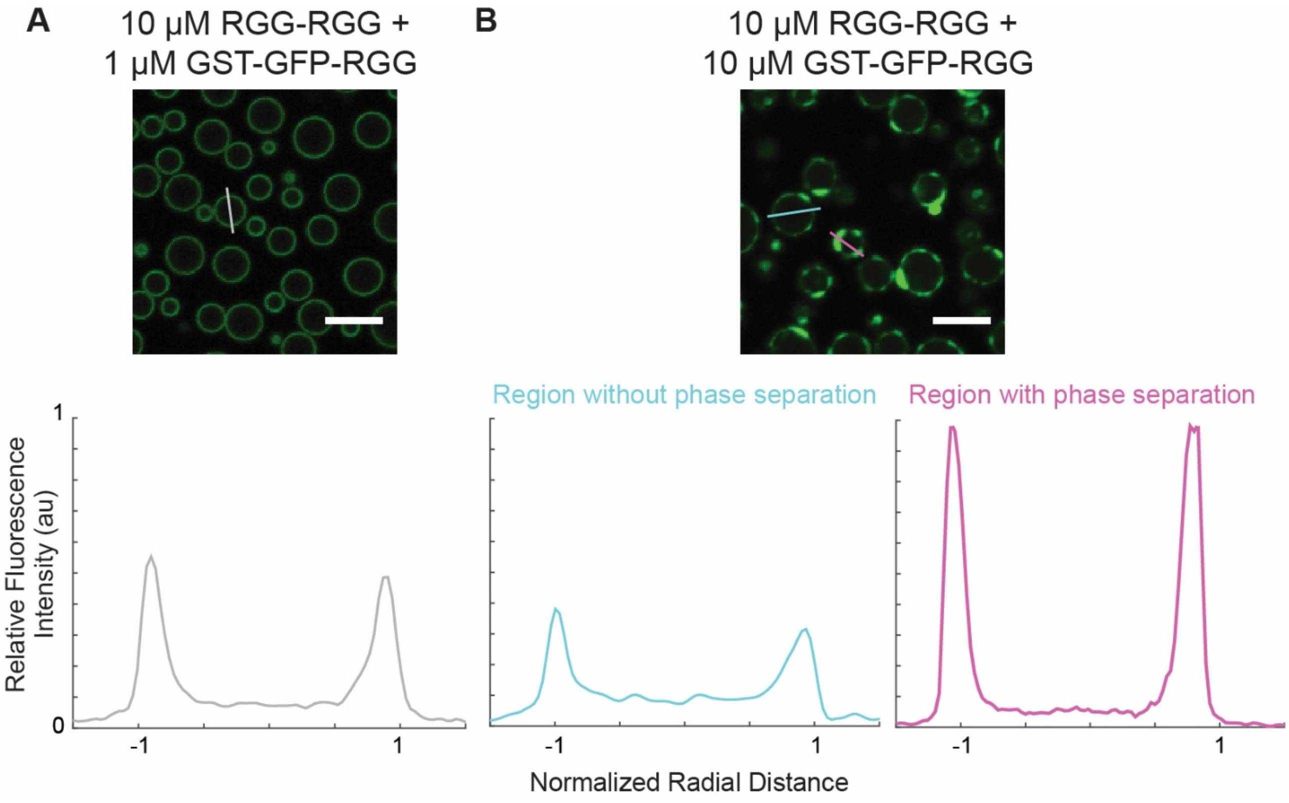

Figure S7: Surfactant protein continues to envelop condensate in conditions with phase separation at the interface. (Top) Representative images and (bottom) average line profiles across RGG-RGG condensates with (A) 1 and (B) 10 μM GST-GFP-RGG. Line profiles represent the average of n = 10 condensates. In (B), the left line profiles (cyan) were drawn in regions of the condensate interface without phase separated GST-GFP-RGG. The right line profiles (magenta) were drawn in regions with phase separated GST-GFP-RGG. Profiles show that GST-GFP-RGG forms a continuous layer surrounding condensates even when phase separation occurs at the interface. Scale bars, 5 μm.

Supplementary Movies

SI Movie 1: Optical tweezer-assisted droplet fusion experiment in a sample containing 10 μM RGG-RGG + 7.5 μM GST-GFP-RGG. Although droplets are approximated using optical tweezers, they do not fuse. Instead, we observe droplets moving vertically on top of each other.
